# Impact of Fixation Pins and Metal Sleeves on the Precision of Guided Endodontics: An Analysis Using Extracted Teeth

**DOI:** 10.4317/jced.62645

**Published:** 2025-09-01

**Authors:** Marco Antonio Zaiden Loureiro, Julio Almeida Silva, Lucas Raineri Capeletti, Emmanuel Joao Nogueira Leal Silva, Maria Luiza Lima Santana, Carlos Estrela, Orlando Aguirre Guedes, Daniel de Almeida Decurcio

**Affiliations:** 1 School of Dentistry, Federal University of Goiás, Goiânia, Goiás, Brazil; 2 School of Dentistry, Alfredo Nasser University Center, Aparecida de Goiânia, Goiás, Brazil; 3 Department of Endodontics, Fluminense Federal University, Niterói, Brazil; 4 Department of Endodontics, Grande Rio University, Rio de Janeiro, Brazil; 5 School of Dentistry, Evangelical University of Goiás, Anápolis, Goiás, Brazil

## Abstract

**Background:**

This study aimed to evaluate the accuracy of guided endodontic access and fiberglass post removal, comparing stabilization methods such as metal sleeves and fixation pins.

**Material and Methods:**

Sixty-four extracted human teeth (32 mandibular molars and 32 single-rooted teeth) were divided into groups based on the use or non-use of fixation pins and metal sleeves. Precision was assessed using pre- and post-procedure CBCT scans to analyze deviations in bur positioning.

**Results:**

For guided endodontic access, significant differences in angular deviation were found between the group using fixation and no sleeves (2.64°) and the group without fixation and sleeves (1.37°) (*P*<0.05). No other significant differences were found in either access or post removal procedures (*P*>0.05). Mean deviations in post removal ranged from 1.98° to 2.15°.

**Conclusions:**

Guided endodontic techniques are highly reliable, with metal sleeves and fixation pins offering no significant improvement in precision.

** Key words:**Guided Endodontics, Dental Post Removal, CBCT Analysis, Endodontic Guide Precision, Endodontic Access Deviation.

## Introduction

Recent advancements in prototyped guides have significantly contributed to the conservative management of complex endodontic cases. Initially, these guides were heralded for their role in locating and accessing calcified canals [1-3]. Their application has since expanded to include meticulous removal of fiberglass posts – a task requiring significant expertise and precision from the practitioner, when performed in a “free-hand” manner [4-6]. Furthermore, prototyped guides have become highly valuable in microsurgical endodontic procedures, assisting in osteotomies and ultrasonic apicectomies [7-9].

Endodontic practices have increasingly borrowed surgical guides from implant dentistry, incorporating software specifically tailored for these applications. As a result, endodontic techniques have repurposed surgical instruments, like drills and metal sleeves, which were not originally designed for endodontic purposes [10,11]. Metal sleeves are posited to play a pivotal role in stabilizing drills during guided procedures. Their dimensions, particularly the internal diameter and height, are carefully matched with the drill size to avoid contact with the guide’s resin. This precise matching minimizes the risk of heat generation and deviations from the digitally planned trajectory [10]. Nonetheless, the practicality of using metal sleeves can be limited in cases with restricted space, such as those involving narrow roots or a limited mouth opening [12,13]. Recent advancements in drill technology have been designed to reduce dental tissue wear, thereby broadening the applications for guided endodontic procedures. These developments include offering a range of drill diameters that are compatible with standard, industrially manufactured sleeves. However, the standardized dimensions of these sleeves may limit compatibility with certain drills, necessitating the customization of guides to accommodate these drills. The ability to customize guide fabrication in planning software allows for adaptation to different drills for each case, potentially eliminating the need for metal sleeves [14].

Bone fixation pins, traditionally utilized in implant dentistry, have been adapted to enhance stability during guided endodontic procedures. By minimizing movement, these pins negate the need for manual stabilization by the clinician or assistant and is claimed to improve procedural efficiency and accuracy [15]. Although their application is commonplace, especially among edentulous patients [16], their use can be uncomfortable for patients, due to surgical requirements. Research suggests that in cases where sufficient adjacent teeth are present to secure the guide, the use of bone fixation might be unnecessary [3,17,18].

Current literature lacks definitive conclusions regarding the essential role of metal sleeves and fixation pins in enhancing the accuracy of guided endodontic procedures. This study was designed to investigate their impact on the precision of creating guided endodontic access and facilitating post removal. The assessment was conducted using Cone Beam Computed Tomography (CBCT) scans, aiming to contribute valuable insights into the efficacy of these tools in endodontic practice. The null hypothesis tested was that the precision of guided endodontic procedures is not significantly affected by the use of metal sleeves and fixation pins.

## Material and Methods

- Sample Selection and Experimental Groups

Ethical approval for this study was obtained from the Local Research Ethics Committee (CAAE 57359422.3.0000.5083). The sample size was calculated based on the standard deviation of angular deviation, 1.81 degrees, observed in the control group as previously reported (19), resulting in a margin of error of 0.887 degrees within a 95% confidence interval requiring the analysis of 16 root canals.

**Figure 1:**
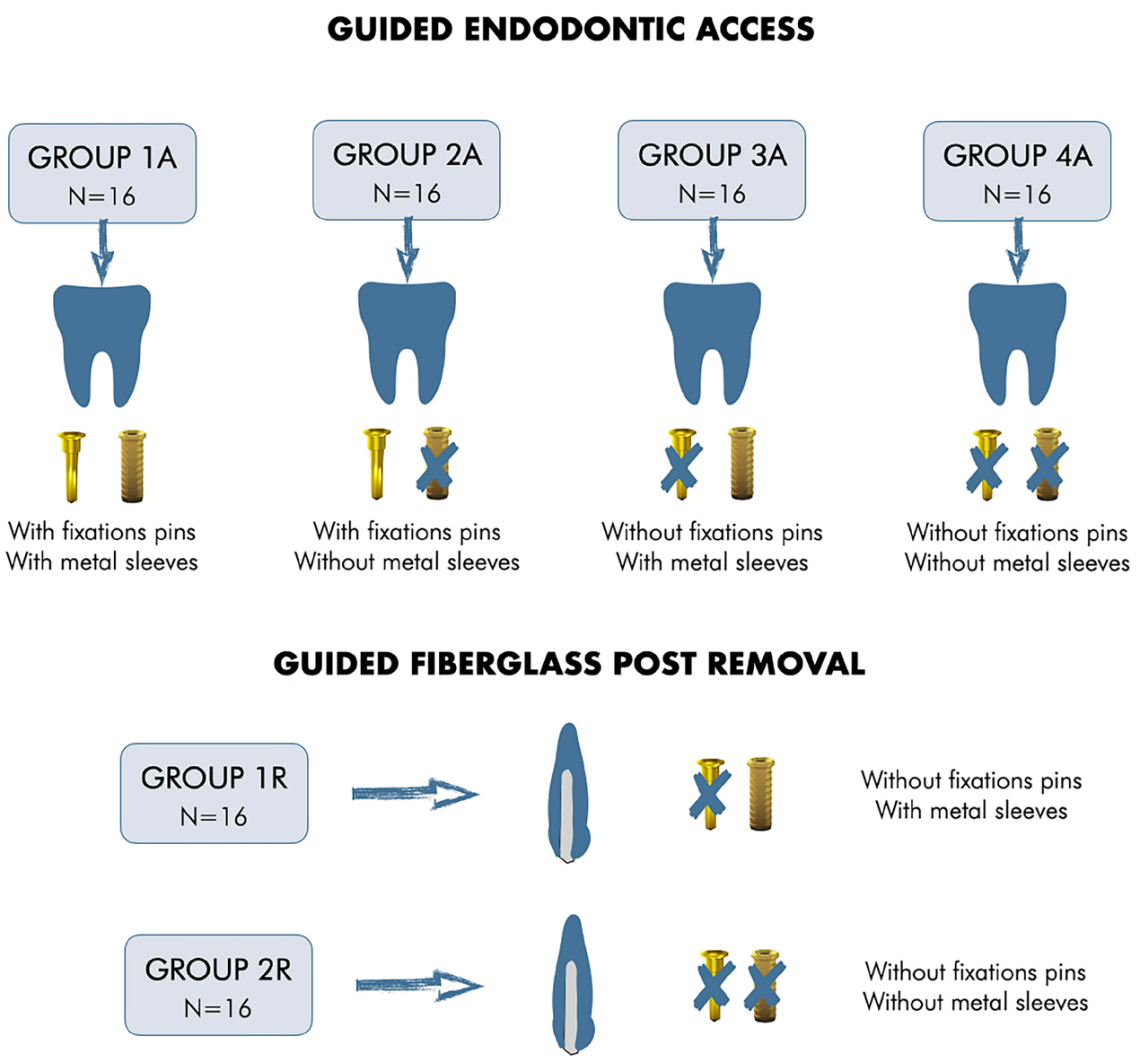
Division of the sample into the respective groups.

Human teeth, comprising 32 single-rooted and 32 mandibular molars, were collected from individuals over 18 years of age, presenting at the Urgency Service of the Faculty of Dentistry, Federal University of Goiás, under approved extraction indications. To remove organic debris, the extracted teeth were immersed in a 5% sodium hypochlorite solution for 30 minutes and subsequently stored in 0.2% thymol solution. Inclusion criteria for the teeth were as follows: a healthy, partially decayed, restored, or fractured crown, alongside roots with complete formation, confirmed through digital periapical radiographs. Exclusion criteria encompassed teeth with root fractures, perforations, or any previous endodontic treatment. The molars were randomized into four groups (*n*=16), and single-rooted teeth were divided into two groups (*n*=16), as depicted in Figure 1.

- Preparation of Samples for Calcified Root Canal Access Guides

In the samples from groups G1A to G4A, procedures commenced with the creation of initial coronal access and the localization of root canal localizations. Subsequently, the coronal chamber was rinsed and dried. A selective etching of the enamel was then performed using 37% phosphoric acid (Condac, FGM, Mogi das Cruzes, São Paulo, Brazil) for 30 seconds, succeeded by a thorough 60 second water rinse. An adhesive system (Single Bond Universal; 3M ESPE, St. Paul, MN, USA) was applied and light-cured for 20 seconds (Valo, Ultradent, South Jordan, UT, USA). To simulate calcification within the pulp chamber, bulk fill resins (Filtek One, 3M Espe, St. Paul, MN, USA) were used to restore the entire pulp chamber. This restoration material was then light cured for 40 seconds.

- Preparation of Samples for Guide-Assisted Fiberglass Post Removal

For groups G1R and G2R, access was performed using spherical drills, with canal preparation 1mm before the apical foramen with an WaveOne Gold Large instrument (45/0.05v; Dentsply Sirona, Ballaigues, Switzerland), with continuous 2.5% sodium hypochlorite irrigation. The root canals were filled using lateral compaction technique with gutta-percha and AH Plus Sealer (Dentsply Sirona, Ballaigues, Switzerland). Preparations for intracanal posts involved Gates-Glidden 1 and 2, and Largo 1 burs (Dentsply Sirona, Ballaigues, Switzerland) to a standard length of 14mm. After saline rinse and drying with paper cones, selective enamel etching was performed using 37% phosphoric acid for 30 seconds, followed by a 60 seconds water rinse. An adhesive was applied in the coronal chamber, then the fiberglass post was treated with 24% hydrogen peroxide for 60 seconds, rinsed, and dried. Silane (Ivoclar Vivadent, Schaan, Liechtenstein) was applied for 60 seconds before cementation with dual self-adhesive cement (SDI, Bayswater, VIC, Australia), light-cured for 60 seconds, and restored with Bulk Fill resin (3M ESPE, St. Paul, MN, USA).

Samples of both calcified root canal access guides and fiberglass post removal groups were embedded in self-curing acrylic resin within mandible-simulating supports.

- CBCT imaging and samples scanning 

Initial CBCT scans for both the calcified root canal access guide and post removal groups were conducted using an Eagle 3D scanner (Dabi Atlante, Ribeirão Preto, SP, Brazil). Samples were positioned to mimic patient head orientation. Imaging parameters included a 0.16 mm voxel size, an 8x8 cm Field of View (FOV), a scan duration of 25.5 seconds, 85 kVp, and a 4 mA current.

Scanning of the samples was performed using the TRIOS scanner (3Shape, Copenhagen, Denmark). Following the scan, 3D images were assessed for quality using the device’s proprietary software. Upon quality confirmation, images were converted to STL format.

- Digital planning and design of the Guides

**Figure 2:**
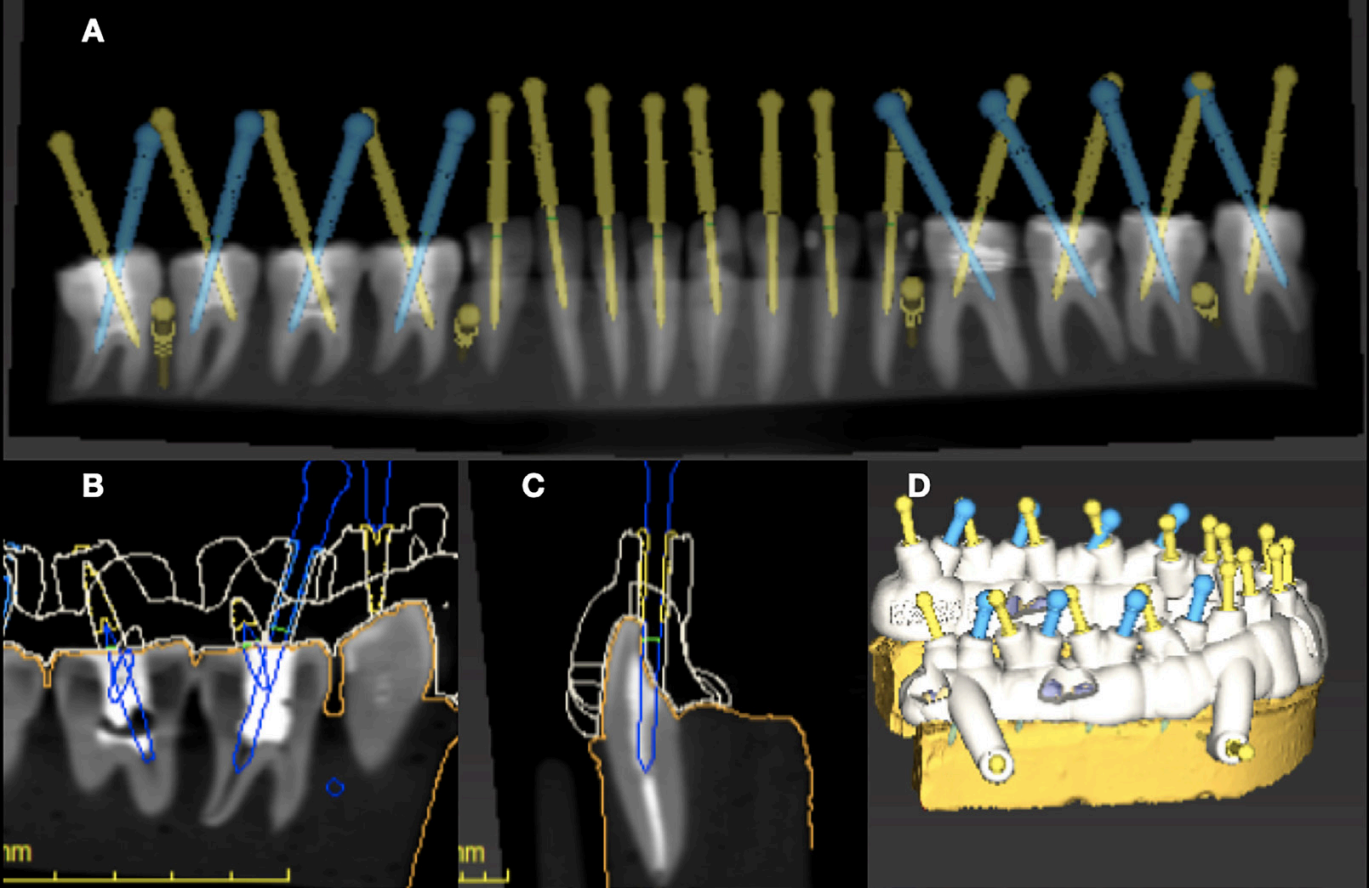
(A) Digital planning of endodontic access for locating canals in molars and removing fiberglass posts in single-rooted teeth; (B) Positioning of the drill for access 3 mm below the root canal entry hole; (C) Positioning of the drill to remove the fiberglass post; (D) Digital planning of guides for access and post removal.

The endodontic guides for this study were meticulously designed using CodiagnostiX software (Straumann, Basel, Switzerland), enabling precise calibration of guide parameters to meet the specific requirements of each test group (Fig. 2). For groups G1A, G3A and G1R, which necessitated maximum stability, metallic sleeves, with an external diameter of 2.5 mm, an internal diameter of 1.5 mm, and a length of 7.5 mm (Neodent, Curitiba, PR, Brazil) were employed. This ensured optimal fit and stability during endodontic access.

Conversely, groups G2A, G4A and G2R, which did not employ metallic sleeves, took a distinct approach. The guides for these groups were designed with a 7.5 mm high access hole and an internal diameter of 1.4 mm, slightly exceeding the 1.3 mm diameter of the drill. This design allowed for smooth drill operation while maintaining guide stability.

For molar endodontics access in groups G1A to G4A, the guides were precisely engineered to permit drilling to a depth of 3 mm below the canal entrance orifice, providing a clear reference point during the procedure (Fig. 2). For the post-removal groups (G1R and G2R), the guides were customized to correspond with the length of the fiberglass posts to facilitate their complete removal (Fig. 2). To enhance stability throughout the access process, the design incorporated eight adjacent teeth for all specified groups (Fig. 2).

- Fabrication of Guides

The manufacturing of the guides followed a stringent protocol to ensure their suitability for clinical application. These guides were produced through 3D printing on a DLP printer (Moonray, SprintRay, Los Angeles, CA, USA) utilizing a resin characterized by its non-toxicity and ability to withstand autoclaving (SprintRay, Los Angeles, CA, USA). Following the printing process, the guides were subjected to a cleaning regimen involving two isopropyl alcohol baths within an ultrasonic chamber, lasting 5 minutes each. The final step involved additional polymerization, conducted in a specialized light-curing oven for resins (Essence Dental) for 30 minutes to solidify and strengthen the material before their use.

-Guided endodontic access and fiberglass post removal

**Figure 3:**
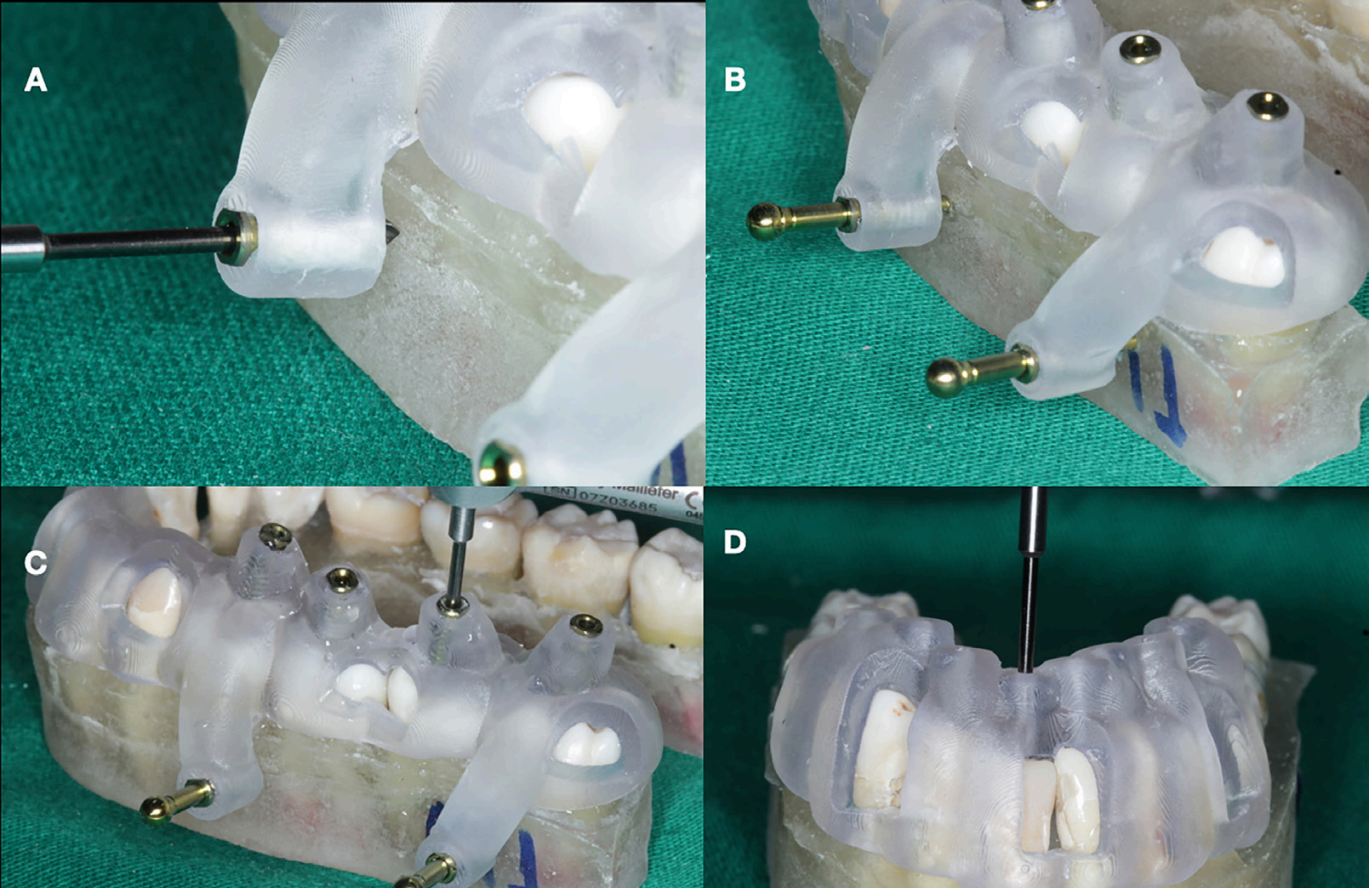
(A) Drilling to use the fixation pins; (B) Fixing pins in position; (C) Guided access with fixing pins and sleeves; (D) Guided Removal of fiber post without retaining pins and sleeves.

After printing, metallic sleeves (Neodent, Curitiba, PR, Brazil) were inserted into the guides for groups G1A, G3A, and G1R, where the use of fixation pins or sleeves was necessitated for access. The accuracy of each guide’s fit was assessed on the teeth through visual inspection windows. In groups G1A and G2A, perforations for fixation pins were made using a drill with a 1.3mm diameter and a length of 20mm (Neodent) (Fig. 3A,B). This drill was also employed access creation in groups G1A, G2A, G3A, and G4A, as well as for removing fiberglass posts in groups G1R and G2R (Fig. 3C,D). An endodontic motor (XSmart Plus, Dentsply Sirona, Ballaigues, Switzerland) set to 800 rpm and a torque of 4 N.cm facilitated the drilling process. Access cavities were prepared and posts removed by employing pecking motions with the drill, advancing 3 mm at each step before retraction for debris removal and irrigation. Upon establishing access, #15 stainless steel instruments (Dentsply Sirona, Ballaigues, Switzerland) were employed, with an operating microscope enhancing canal mapping and exploration.

- Access accuracy analysis

**Figure 4:**
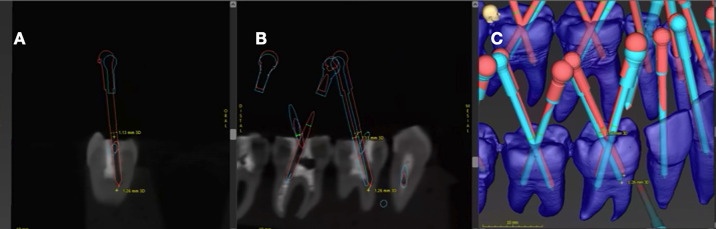
(A) Superposition of planned trajectories in a CBCT coronal section; (B) Sagittal section of measurement of CBCT deviations; (C) Volumetric reconstruction of trajectory deviation analyses.

After the guided step, the samples underwent new CBCT scans using the original settings. The resulting images were uploaded into the CodiagnostiX software for detailed analysis. A trained operator manually delineated the drill path on these images, which were then superimposed over the initial CBCT scans within the software. Linear deviations at the drill tip and angular deviations were qualified using the software’s measurements tools, adhering to the measurement precision protocols established by Chaves *et al*., [19] (Fig. 4).

- Statistical analysis

Data normality was assessed using the Kolmogorov-Smirnov test. The comparison between groups was analyzed using the Student t-test for independent samples, Analysis of Variance (ANOVA), or Kruskal-Wallis tests. A significance level of *p* < 0.05 was considered statistically significant. Statistical analysis was conducted using the Statistical Package for the Social Sciences, version 20 (SPSS, Chicago, IL).

## Results

 Table 1 and  Table 2 present the mean and standard deviation for the three-dimensional deviations measured in both degrees and millimeters. These Tables also detail the millimeter deviations for the distal, buccal, and apical measurements across the various groups. A statistical difference was found between groups G2A and G4A regarding the degree of deviations (*P*<0.05). However, no significant statistical differences were detected in the deviation measurements, neither in degrees nor millimeters, among the other groups for both guided access and fiberglass post removal. No accidents or perforations were recorded.

## Discussion

The research focused on evaluating the effectiveness of guided endodontics in two primary areas: accessing calcified canals and removing fiberglass posts. Through an in-depth comparison across various protocols and group configurations—specifically, those with and without the use of metal sleeves and fixation pins—the study not only aimed to enhance the understanding of guided endodontic procedures but also sought to expand the range of indications for guided access. Additionally, it strived to establish standardized procedures for these techniques in simulated clinical settings.

El Kholy *et al*. [20] investigated the impact of the number of adjacent teeth used for guide stabilization on the accuracy of implant placement via guided surgeries. Their research concluded that the precision of implant placement did not significantly vary between guides supported by a bilateral arrangement and those supported by only four teeth. Contrasting with their findings, the current study standardized the use of guides spanning a minimum of eight teeth to facilitate direct comparisons concerning the use of fixation pins and metallic sleeves. According to  Table 1, discrepancies between planned trajectories and actual outcomes were noted across all groups. However, statistically significant differences were only found between the groups in terms of angular deviations, specifically between the group utilizing fixation pins without sleeves and the group without either, with the latter demonstrating the lowest deviation values.

Tavares *et al*. [15] highlighted the challenges inherent to guided endodontics, particularly stressing the need for well-defined fixation protocols for guides in bone. While there has been advocacy for fixation pins to minimize deviations during clinical procedures, research demonstrates that guided endodontics, even in the absence of these pins, is safe, efficient, and conservative for canal localization [3,17,18], consistent with the outcomes of the current study. In the present investigation, groups undergoing accessed without metal sleeves demonstrated reduced variations. Specifically, the metal sleeves from the Neodent system feature an internal diameter of 1.5mm, compared to the drill diameter of 1.3mm. The observed enhanced stability during access in groups devoid of metal sleeves can be attributed to the narrower diameter differential, implying that forgoing sleeves might improve stability. These findings align with earlier research [12,13], further substantiating the potential benefits of sleeveless guided endodontic procedures. Nonetheless, it remains imperative for future studies to explore various sizes of metal sleeves and drills to ascertain if these outcomes can be replicated. Investigating a broader spectrum of diameter differences could yield insights into optimizing stability and precision in guided endodontic procedures. This approach would help validate the current findings and potentially refine the selection criteria for endodontic tools, ensuring the highest standards of procedural efficacy and patient care.

Analyzing deviations toward the apical direction is essential for the planning of guided endodontic access. Findings from this investigation reveal that across all study groups, actual trajectories consistently fell short of the planned depths. This discrepancy suggests that plans should account for an additional 1mm beyond the intended target depth to compensate for these deviations, which ranged from -0.42 ± 0.57 mm to -0.60 ± 0.47 mm in the guided access groups and between -0.98 ± 0.53 mm and -0.81 ± 0.56 mm in the fiberglass post removal groups. This study’s observations are in line with outcomes reported previously [14,19], reinforcing the practicality and precision of guided endodontic access. Nonetheless, the reliance on extracted human teeth as the basis for this study introduces a constraint to the direct applicability of these results to clinical settings. Therefore, further clinical research is essential to fully validate the findings and ensure their relevance to actual endodontic procedures.

Moreover, while deviations were noted across all groups, the mean deviations from the planned and the executed procedures varied from 0.91 to 1.11 mm for endodontic accesses and from 1.11 and 1.36 mm for post removal. These minor discrepancies affirm the reliability of guided endodontic techniques, suggesting a recommended safety margin of around 1mm for guided accesses and approximately 1.5mm for guided post removals. This margin provides practitioners with a level of confidence when performing these techniques, acknowledging a small but manageable level of variation from planned outcomes.

This study, while providing valuable insights into the precision of guided endodontic procedures, has some limitations. The use of extracted human teeth, although beneficial for controlled experimental conditions, may not fully replicate the clinical scenario, including the impact of patient movement and soft tissue presence. Furthermore, the study employed resins to simulate calcification, which may not accurately reflect the complex characteristics of real calcified tissues. Additionally, the study’s design does not account for the variability in clinical practice, such as differences in operator skill and experience. Future research should consider these factors and include clinical trials to better understand the real-world applicability of the findings. Moreover, the study’s focus on specific types of metal sleeves and fixation pins may limit the generalizability of the results to other systems and materials. Expanding the range of tools and techniques evaluated would enhance the robustness and applicability of the study’s conclusions. Moreover, variables such as restorative materials causing CBCT artifacts, operational positioning, patient movement, digital guide stabilization by the operator, and the number of adjacent teeth for stabilization can influence outcomes. Hence, additional clinical studies are warranted to further validate the laboratory model’s findings.

## Conclusions

Although all groups exhibited deviations, the difference between the planned and actual outcomes of the guided endodontic procedures was minimal, underscoring the technique’s reliability. Notably, the use of metal sleeves and fixation pins did not markedly improve procedural precision.

## Figures and Tables

**Table 1:** Mean and standard deviation of the deviation in degrees, deviation in mm, distal tip, buccal tip and apical tip of groups 1A, 2A, 3A and 4A.

Guided endodontic access	Group 1A with fixation with sleeve	Group 2A with fixation without sleeve	Group 3A no fixation with sleeve	Group 4A no fixation without sleeve	P
Deviation in degrees	1.56 ±1.10AB	2.64 ±1.30A	1.92 ±1.30AB	1.37 ±0.92B	0.035
Deviation in mm	0.91 ±0.56A	1.01 ±0.43A	1.11 ±0.49A	1.08 ±0.70A	0.745
Distal	-0.16 ±0.48A	0.15 ±0.48A	-0.006 ±0.54A	-0.23 ±0.85A	0.330
Buccal	0.31 ±0.64A	0.51 ±0.45A	0.60 ±0.60A	0.22 ±0.74A	0.280
Apical	-0.54 ±0.36A	-0.60 ±0.47A	-0.42 ±0.57A	-0.47 ±0.37A	0.724

Different letters on the same line indicate a significant difference (*p* <0.05).

**Table 2:** Mean and standard deviation of deviation in degrees, deviation in mm, distal tip, buccal tip and apical tip of groups 1R and 2R.

Post Removal Guides	Group 1R no fixation with sleeve	Group 2R no fixation without sleeve	P
Deviation in degrees	2.15 ±1.32A	1.98 ±1.40A	0.768*
Deviation in mm	1.36 ±0.58A	1.10 ±0.57A	0.214*
Distal	-0.08 ±0.49A	-0.18 ±0.42A	0.525*
Buccal	0.75 ±0.41A	0.51 ±0.35A	0.085*
Apical	-0.98 ±0.53A	-0.81 ±0.56A	0.383*

*T-test for independent samples.

## Data Availability

The datasets used and/or analyzed during the current study are available from the corresponding author.

## References

[1] Krastl G, Zehnder MS, Connert T, Weiger R, Kühl S (2016). Guided Endodontics: a novel treatment approach for teeth with pulp canal calcification and apical pathology. Dent Traumatol.

[2] Connert T, Zehnder MS, Weiger R, Kühl S, Krastl G (2017). Microguided Endodontics: accuracy of a miniaturized technique for apically extended access cavity preparation in anterior teeth. J Endod.

[3] Loureiro MAZ, Silva JA, Chaves GS, Capeletti LR, Estrela C, Decurcio DA (2021). Guided endodontics: the impact of new technologies on complex case solution. Aust Endod J.

[4] Schwindling FS, Tasaka A, Hilgenfeld T, Rammelsberg P, Zenthöfer A (2020). Three-dimensional-guided removal and preparation of dental root posts-concept and feasibility. J Prosthodont Res.

[5] Maia LM, Bambirra Júnior W, Toubes KM, Moreira Júnior G, Machado VC, Parpinelli BC (2022). Endodontic guide for the conservative removal of a fiber-reinforced composite resin post. J Prosthet Dent.

[6] Krug R, Schwarz F, Dullin C, Leontiev W, Connert T, Krastl G (2024). Removal of fiber posts using conventional versus guided endodontics: a comparative study of dentin loss and complications. Clin Oral Investig.

[7] Ahn S, Kim N, Kim S, Karabucak B, Kim E (2018). Computer-aided Design/Computer-aided Manufacturing-guided Endodontic Surgery: guided osteotomy and apex localization in a mandibular molar with a thick buccal bone plate. J Endod.

[8] Ackerman S, Aguilera FC, Buie JM, Glickman GN, Umorin M, Wang Q (2019). Accuracy of 3-dimensional-printed Endodontic Surgical Guide: a human cadaver study. J Endod.

[9] Chaves GS, Capeletti LR, Miguel JG, Loureiro MAZ, Silva EJNL, Decurcio DA (2022). A Novel Simplified Workflow for Guided Endodontic Surgery in Mandibular Molars with a Thick Buccal Bone Plate: a case report. J Endod.

[10] Decurcio DA, Bueno MR, Silva JA, Loureiro MAZ, Sousa-Neto MD, Estrela C (2021). Digital Planning on Guided Endodontics Technology. Braz Dent J.

[11] Connert T, Weiger R, Krastl G (2022). Present status and future directions - Guided endodontics. Int Endod.

[12] Torres A, Lerut K, Lambrechts P, Jacobs R (2021). Guided Endodontics: Use of a Sleeveless Guide System on an Upper Premolar with Pulp Canal Obliteration and Apical Periodontitis. J Endod.

[13] Torres A, Dierickx M, Coucke W, Pedano MS, Lambrechts P, Jacobs R (2023). In vitro study on the accuracy of sleeveless guided endodontics and treatment of a complex upper lateral incisor. J Dent.

[14] Pires CRF, Souza-Gabriel AE, Pelozo LL, Cruz-Filho AM, Sousa-Neto MD, Silva RG (2022). Guided endodontics of calcified canals: The drilling path of rotary systems and intracanal dentin wear. Aust Endod J.

[15] Tavares WLF, Pedrosa NOM, Moreira RA, Braga T, Machado VC, Ribeiro Sobrinho AP (2022). Limitations and Management of Static-guided Endodontics Failure. J Endod.

[16] Pessoa R, Siqueira R, Li J, Saleh I, Meneghetti P, Bezerra F (J Prosthodont 2022). The Impact of Surgical Guide Fixation and Implant Location on Accuracy of Static Computer-Assisted Implant Surgery.

[17] Zehnder MS, Connert T, Weiger R, Krastl G, Kühl S (2020). Guided endodontics: accuracy of a novel method for guided access cavity preparation and root canal location. Int Endod J.

[18] Loureiro MAZ, Elias MRA, Capeletti LR, Silva JA, Siqueira PC, Chaves GS (2020). Guided Endodontics: volume of dental tissue removed by guided access cavity preparation - an ex vivo study. J Endod.

[19] Chaves GS, Silva JA, Capeletti LR, Silva EJNL, Estrela C, Decurcio DA (2023). Guided Access Cavity Preparation Using a New Simplified Digital Workflow. J Endod.

[20] El Kholy K, Lazarin R, Janner SFM, Faerber K, Buser R, Buser D (2019). Influence of surgical guide support and implant site location on accuracy of static Computer-Assisted Implant Surgery. Clin Oral Implants Res.

